# DHT and Insulin Upregulate Secretion of the Soluble Decoy Receptor of IL-33 From Decidualized Endometrial Stromal Cells

**DOI:** 10.1210/endocr/bqad174

**Published:** 2023-11-16

**Authors:** Daniel Salamon, Dorina Ujvari, Anton Hellberg, Angelica Lindén Hirschberg

**Affiliations:** Department of Women's and Children's Health, Karolinska Institute, SE-171 64 Stockholm, Sweden; Department of Women's and Children's Health, Karolinska Institute, SE-171 64 Stockholm, Sweden; Department of Microbiology, Tumor and Cell Biology, National Pandemic Centre, Centre for Translational Microbiome Research, Karolinska Institute, SE-171 64 Stockholm, Sweden; Department of Women's and Children's Health, Karolinska Institute, SE-171 64 Stockholm, Sweden; Department of Women's and Children's Health, Karolinska Institute, SE-171 64 Stockholm, Sweden; Department of Gynecology and Reproductive Medicine, Karolinska University Hospital, SE-171 76 Stockholm, Sweden

**Keywords:** IL-33, sST2, DHT, insulin, endometrium, decidualization

## Abstract

Interleukin 33 (IL-33) signaling regulates most of the key processes of pregnancy, including decidualization, trophoblast proliferation and invasion, vascular remodeling, and placental growth. Accordingly, dysregulation of IL-33, its membrane-bound receptor (ST2L, transducer of IL-33 signaling), and its soluble decoy receptor (sST2, inhibitor of IL-33 signaling) has been linked to a wide range of adverse pregnancy outcomes that are common in women with obesity and polycystic ovary syndrome, that is, conditions associated with hyperandrogenism, insulin resistance, and compensatory hyperinsulinemia. To reveal if androgens and insulin might modulate uteroplacental IL-33 signaling, we investigated the effect of dihydrotestosterone (DHT) and/or insulin on the expression of *ST2L* and *sST2* (along with the activity of their promoter regions), *IL-33* and *sIL1RAP* (heterodimerization partner of sST2), during *in vitro* decidualization of endometrial stromal cells from 9 healthy women. DHT and insulin markedly upregulated sST2 secretion, in addition to the upregulation of its messenger RNA (mRNA) expression, while the proximal *ST2* promoter, from which the *sST2* transcript originates, was upregulated by insulin, and in a synergistic manner by DHT and insulin combination treatment. On the other hand, *sIL1RAP* was slightly downregulated by insulin and *IL-33* mRNA expression was not affected by any of the hormones, while *ST2L* mRNA expression and transcription from its promoter region (distal ST2 promoter) could not be detected or showed a negligibly low level. We hypothesize that high levels of androgens and insulin might lead to subfertility and pregnancy complications, at least partially, through the sST2-dependent downregulation of uteroplacental IL-33 signaling.

Interleukin 33 (IL-33) is a primarily nuclear localized alarmin, which is constitutively expressed in various cell types including epithelial, endothelial, and fibroblast cells, and released as a cytokine usually in response to inflammation, tissue damage or nonapoptotic cell death. IL-33 binds to its specific primary receptor ST2 (also known as interleukin 1 receptor like 1 [IL1RL1]). The *ST2* gene has 2 alternative promoters followed by different noncoding first exons, which are spliced to the common second exon. Alternative splicing of the downstream exons give rise to the 2 main *ST2* isoforms: a long transmembrane form (*ST2L*), which activates downstream signaling pathways on IL-33 binding, and a short, soluble decoy receptor (*sST2*), which binds IL-33 with similarly high affinity and specificity, but incapable of signaling and therefore acts as a molecular trap of IL-33. *ST2L* is mainly transcribed from the distal, while *sST2* mainly from the proximal promoter region, in a cell type–specific manner. Binding of IL-33 to ST2L leads to the recruitment of the coreceptor IL-1 receptor accessory protein (IL1RAP), forming a heterodimeric complex, essential for signaling through ST2L. Furthermore, the soluble form of IL1RAP (sIL1RAP) enhances the ability of sST2 to inhibit IL-33 activity (1‐3).

In the human uteroplacental unit, IL-33 expression has been shown in decidual and placental macrophages and endothelial cells, and decidual stromal and epithelial cells, while ST2L expression was detected in proliferative and invasive trophoblasts, decidual stromal, natural killer (NK) and B cells (4‐9). Several lines of evidence suggest that, acting through trophoblasts, decidual stromal, and uterine NK cells, IL-33 signaling regulates most of the key processes of pregnancy, including decidualization, trophoblast invasion, uteroplacental T_H_1/T_H_2 shift, vascular remodeling, and placental growth (5‐10).

Insulin resistance and compensatory hyperinsulinemia, in addition to hyperandrogenism, common clinical conditions in polycystic ovary syndrome (PCOS) and obesity, are associated with gestational hypertension, preeclampsia, miscarriage, and recurrent pregnancy loss (11‐16). Although several further lines of evidence also suggest that the increased production of insulin and androgens might play a key role in the pathophysiology of these pregnancy complications, the molecular mechanisms are still unclear.

Since decidual stromal cells play a central role in human uteroplacental IL-33 signaling (4, 6‐9), we analyzed the effects of dihydrotestosterone (DHT) and insulin, separately and in combination on the expression of *IL-33*, *ST2L*, and *sST2* in decidualizing primary human endometrial stromal cells (ESCs) *in vitro*, with the aim to reveal a novel potential molecular link between high insulin and androgen levels and pregnancy complications.

## Materials and Methods

### Participants

Regularly cycling, healthy volunteers (n = 9) underwent collection of endometrial biopsy under local anesthesia using a suction curette (Pipet Curet, CooperSurgical) in the proliferative phase of the menstrual cycle at cycle day 5 to 9. All women were between ages 18 and 35 years and had a body mass index between 19 and 28. Exclusion criteria were hormonal medication within 3 months prior to biopsy sampling, smoking, endocrine disorder, current chronic disease, or continuous medication. They gave their written informed consent and the regional ethical committee in Stockholm approved the study (DNR 2018/2199-31).

### Isolation of Human Endometrial Stromal Cells and Culture Conditions

Isolation of human ESCs was carried out as described previously (17). ESCs were seeded to 6-well Costar plates (Sigma-Aldrich) at a density of 10^5^/well and cultured in Dulbecco’s modified Eagle’s medium/F12-Glutamax medium (Thermo Fisher Scientific) supplemented with 10% heat-inactivated fetal bovine serum (HI-FBS) (Sigma-Aldrich) and 0.2% penicillin-streptomycin (Sigma-Aldrich) until 80% to 90% confluency. ESCs were decidualized *in vitro* using 1 μM medroxyprogesterone-17-acetate (MPA) (Sigma-Aldrich) and 0.5 mM dibutyryl–cyclic adenosine monophosphate (db-cAMP) (Sigma-Aldrich) in phenol-red free Dulbecco’s modified Eagle’s medium/F12 (Thermo Fisher Scientific) supplemented with 2% charcoal-stripped FBS (Sigma-Aldrich, USA) 0.2% penicillin-streptomycin (Sigma-Aldrich), in the presence or absence of 100 nM insulin (Sigma-Aldrich), 1 μM DHT (Sigma-Aldrich) or the combination of insulin and DHT for 6 days. The culture media was changed after 3 days. We used DHT instead of testosterone, as DHT has a 2-fold higher affinity and 5-fold decreased dissociation rate to the androgen receptor compared with testosterone (18). Furthermore, testosterone can be converted to estradiol by aromatase, while DHT is nonaromatizable (19).

### RNA Isolation, Complementary DNA Synthesis, and Reverse Transcription–Polymerase Chain Reaction

Total RNA was extracted using Quick-RNA Miniprep Kit (Zymo Research) and subjected to complementary DNA (cDNA) synthesis using a SuperScript VILO cDNA Synthesis Kit (Thermo Fisher Scientific). Gene expression levels were determined using the SybrGreen method. Ribosomal protein L13A (*RPL13A*) was used as a housekeeping gene to normalize gene expression levels. Gene expression levels were analyzed with the ^ΔΔ^C_t_ method. The employed oligonucleotides (Sigma-Aldrich) are listed in Supplementary Table 1 (20). All determinations were performed in triplicate.

### Enzyme-linked Immunosorbent Assay

Secreted ST2 protein produced by the decidualizing stromal cells in the absence or presence of DHT, and/or insulin was measured from conditioned media using the human ST2/IL-33R Quantikine enzyme-linked immunosorbent assay (ELISA) kit (R&D Systems; RRID:AB_3064899) according to the manufacturer's instruction. Briefly, stromal cells were decidualized with 1 μM MPA and 0.5 mM db-cAMP in the presence or absence of 1 μM DHT and/or 100 nM insulin for 6 days. Media and treatment were renewed after 3 days, and the conditioned media collected on the sixth day were processed for enzyme-linked immunosorbent assay. The experiment was performed from treatments using 9 healthy volunteers in duplicates.

### Statistical Analysis

Statistical analysis was performed with the R studio software (version 1.4.1106). Two-way analysis of variance for repeated measurements was used with the within-group factors DHT (yes/no), insulin (yes/no), and the interaction DHT × insulin. Considering the limited sample size and the high risk of type II error (false-negative results), we performed simple main effect tests when interactions corresponded to *P* less than .15. Paired *t* test was used to analyze the effect of *in vitro* decidualization on the measured factors. All the data were log-transformed because of skewness. For data visualization, the R package ggplot2 was applied (21). *P* less than .05 was considered statistically significant.

## Results

### Relative Messenger RNA Expression and Protein Levels of the Components of the IL-33/sST2 Signaling Pathway in Response to In Vitro Decidualization

*In vitro* decidualization significantly increased sST2 protein secretion (*P* < .001), mRNA expression originating from the proximal *ST2* promoter (*P* < .01), and *sIL1RAP* mRNA expression (*P* < .05), while the change of *sST2* and *IL-33* mRNA expression remained insignificant (Supplementary Fig. S1 (20)). *ST2L* mRNA was undetectable or showed a negligibly low level in nondecidualized or decidualized ESCs (data not shown).

### Relative mRNA Expression and Protein Levels of the Components of the Interleukin 33/Soluble Interleukin 33Receptor Signaling Pathway in Response to Dihydrotestosterone, Insulin, and Their Combination in Decidualized Primary Human Endometrial Stromal Cells In Vitro

#### Long transmembrane form of IL-33 receptor messenger RNA expression levels

*ST2L* mRNA was undetectable or showed a negligibly low level in the untreated or any of the treated samples of decidualizing ESCs (data not shown).

#### Messenger RNA expression originating from the distal interleukin-33 receptor promoter (encoding mainly the long transmembrane form of the interleukin-33 receptor)

Transcript originating from the distal *ST2* promoter could not be detected in the untreated or any of the treated samples of decidualizing ESCs (data not shown).

#### Soluble interleukin-33 receptor messenger RNA and secreted protein levels

**Figure 1.**
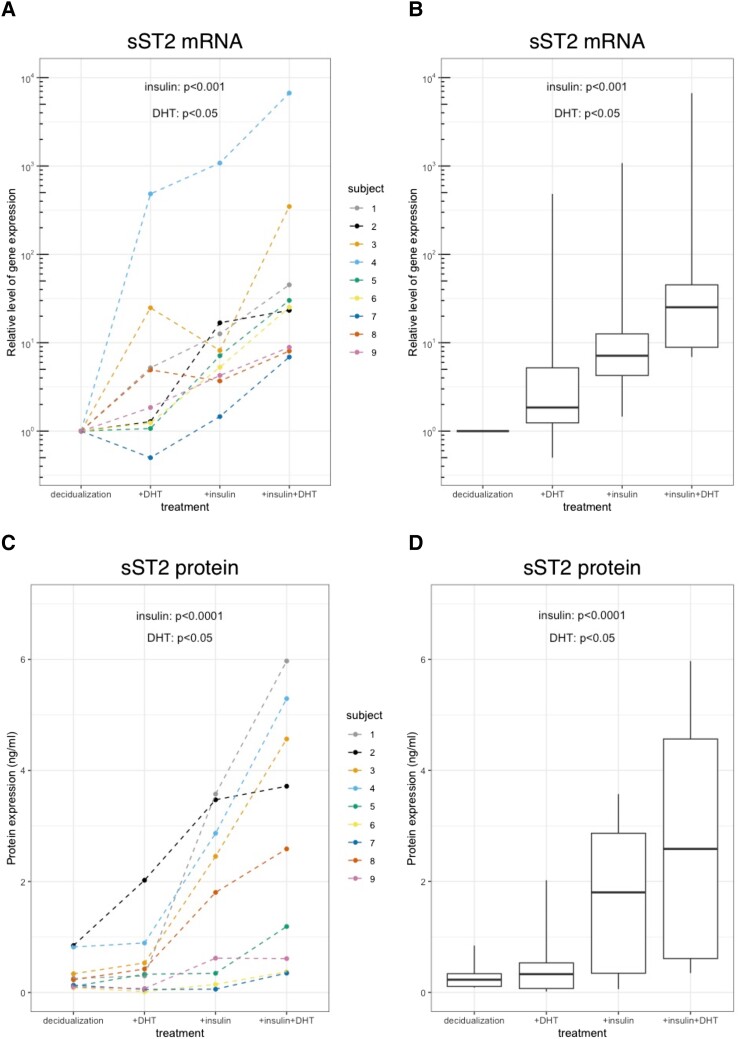

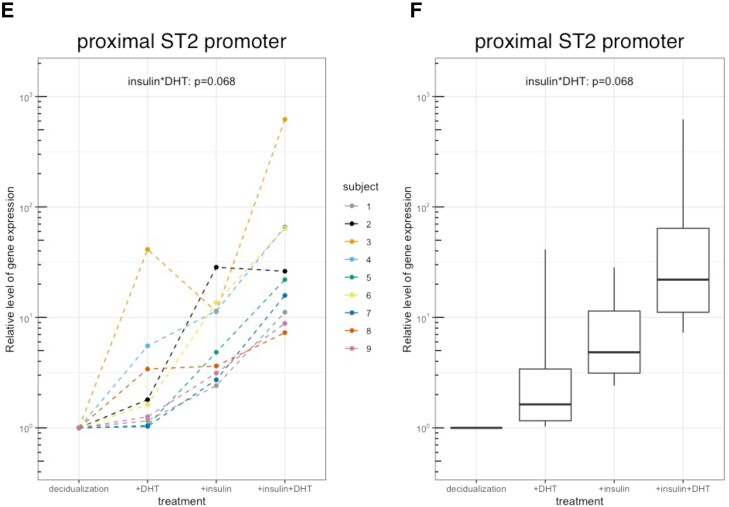
Effects of dihydrotestosterone (DHT) and insulin on short, soluble IL-33 receptor (*sST2*) messenger RNA (mRNA) expression, sST2 protein secretion, and proximal *ST2* promoter activity in *in vitro* decidualized human endometrial stromal cells (ESCs). A and B, Relative levels of *sST2* mRNA expression after 6 days in response to DHT and/or insulin in decidualized *in vitro* (treated with medroxyprogesterone-17-acetate [MPA] and dibutyryl–cyclic adenosine monophosphate [db-cAMP]) ESCs of 9 healthy volunteers. Main effect of DHT: *P* less than .05; main effect of insulin: *P* less than .001. C and D, sST2 protein expression in the conditioned media of *in vitro* decidualized (treated with MPA and db-cAMP) ESCs of 9 healthy volunteers after 6 days in response to DHT and/or insulin. Main effect of DHT: *P* less than .05; main effect of insulin: *P* less than .0001. E and F, Relative levels of *ST2* mRNA originating from the proximal *ST2* promoter after 6 days in response to DHT and/or insulin in decidualized *in vitro* (treated with MPA and db-cAMP) ESCs of 9 healthy volunteers. Interaction effect between insulin and DHT: *P* = .068. Data are shown as individual values (A, C, E) and as a box plot, representing median, interquartile ranges, and ranges (B, D, and F).

Both DHT and insulin increased *sST2* mRNA levels (*P* < .05 and *P* < .001, respectively) and sST2 protein secretion (*P* < .05 and *P* < .0001, respectively). There was no significant interaction between DHT and insulin (Figs. 1A, 1B, 1C, and 1D and Table 1). In most of the samples, insulin or the combined treatment induced secretion of sST2 protein in nanogram per milliliter (ng/mL) range in the conditioned media.

**Table 1.** Comparison of relative gene expression levels and protein levels in the presence or absence of dihydrotestosterone and insulin in decidualized *in vitro* endometrial stromal cells

Gene/Protein	Decidual	DHT	Insulin	DHT + Insulin	Interaction and main effect	Post hoc effects
sST2 mRNA	1.63 (0.05-23.69)	13.28 (0.26-38.71)	11.63 (0.63-398.08)	59.87 (2.26-551.77)	A^*^, B^***^	
proximal ST2 promoter	6.86 (1.00-31.02)	12.59 (1.16-54.51)	18.66 (2.41-422.33)	108.47 (11.14-1985.08)	1^(*P* = .068)^	a^***^, c^**^, d^***^
sIL1RAP mRNA	1.21 (0.89-2.04)	1.20 (0.92-1.75)	0.92 (0.67-1.23)	0.80 (0.59-1.21)	B^**^	
IL-33 mRNA	0.48 (0.23-12.87)	0.31 (0.12-2.74)	0.57 (0.31-7.21)	0.97 (0.17-7.42)		
sST2 protein	0.23 (0.09-0.85)	0.33 (0.02-2.02)	1.80 (0.06-3.57)	2.59 (0.35-5.97)	A^*^, B^****^	

Data are median and ranges (minimum-maximum). Values of *sST2*, proximal *ST2* promoter, *sIL1RAP*, and *IL-33* are relative mRNA expression levels compared with those in stromal cells. Values of sST2 protein expression levels are in ng/mL. 1 = Interaction between insulin and DHT, A = main effect of DHT, B = main effect of insulin. Post hoc test: a = insulin vs decidual, b = insulin + DHT vs insulin, c = insulin + DHT vs DHT.

Abbreviations: DHT, dihydrotestosterone; IL, interleukin; mRNA, messenger RNA; sST2, soluble IL-33 receptor.

^*^ *P* < .05.

^**^ *P* < .01.

^***^ *P* < .001.

^****^ *P* < .0001.

#### Messenger RNA expression originating from the proximal interleukin-33 receptor promoter (encoding mainly soluble interleukin-33 receptor)

There was an interaction between DHT and insulin (*P* = .068), and the post hoc test showed higher mRNA expression originating from the proximal *ST2* promoter by the combined DHT and insulin treatment compared with DHT (*P* = .000) or insulin alone (*P* = .003). Furthermore, the effect of DHT did not reach the level of statistical significance (*P* = .052), while insulin enhanced proximal promoter region expression compared with no treatment (*P* = .000) (Fig. 1E and 1F and Table 1).

#### Soluble IL1RAP messenger RNA expression levels

DHT had no effect, whereas insulin decreased *sIL1RAP* mRNA expression (*P* = .003) (Supplementary Fig. S2 (20) and Table 1). Furthermore, there was no interaction between the two treatments.

#### Interleukin-33 messenger RNA expression levels

There was no significant main effect or interaction between DHT and insulin on *IL-33* gene expression (Supplementary Fig. S3 (20) and Table 1).

## Discussion

In this study, we demonstrated for the first time that both DHT and insulin markedly upregulated *sST2* mRNA expression and sST2 protein secretion in decidualized primary human ESCs *in vitro*. In addition, the proximal *ST2* promoter, from which the *sST2* transcript originates, was upregulated by insulin, and in a synergistic manner by DHT and insulin in combination. On the other hand, *sIL1RAP* was significantly downregulated by insulin and *IL-33* mRNA expression was not affected by any of the hormones, while *ST2L* mRNA expression and transcription from the distal *ST2* promoter could not be detected or showed a negligibly low level in all the treatments.

The formation of the specialized endometrial niche essential for the successful completion of implantation starts with decidualization of the endometrium. This process includes the differentiation of ESCs to acquire a secretory phenotype, in parallel with the recruitment of a large number of various immune cells (including NK cells, macrophages, T cells, B cells, and dendritic cells) to the endometrium (4, 22, 23). These cells, at the fetomaternal interface, play an essential role both in immunological aspects and several key biological processes of pregnancy, such as trophoblast invasion and vascular remodeling. Accordingly, the composition and activity of these cells must shift during pregnancy (22, 23), and this is, at least partially, driven by the sequential activation of the IL-33/ST2L/sST2 pathway (6, 10). For example, the dominance of a proinflammatory (T_H_1-type) microenvironment is essential for optimal decidualization, implantation, placentation, and the reparation of the damage caused by the invading blastocyst, while, from the second trimester, an anti-inflammatory (T_H_2-type) immune activity is necessary for optimal fetal growth. Shortly before parturition, the proinflammatory T_H_1 starts dominating again and initiates labor (22‐24). In line with the proposed key role of IL-33 in pregnancy, a recent publication showed that maternal IL-33 deficiency in mice inhibits uterine T_H_2 responses by group 2 innate lymphoid cells, CD4+ T cells, and M2-polarized macrophages, while it concomitantly impairs decidualization and implantation chamber formation and induces abnormal decidual vascularization and spiral artery remodeling, leading to delayed early embryo development, increased resorption rates, and impaired fetal and placental growth during late pregnancy (10).

Previous studies demonstrated that ex vivo IL-33 treatment increased the proliferation of primary human trophoblast cells (7) and the proliferation and invasiveness of primary human decidual stromal cells (9), and the addition of recombinant human sST2 completely blocked all of these effects (7, 9). Furthermore, decidual stromal cell–derived IL-33 increased anti-inflammatory (T_H_2-type) cytokine production and inhibited cytotoxicity of primary human decidual NK cells in an ST2L-dependent manner (8). Our observations suggest that high uteroplacental levels of DHT and insulin might inhibit these IL-33 effects through the upregulation of sST2 secretion from decidualizing ESCs.

Contrary to the upregulation of *sST2* expression, the mRNA level of its heterodimerization partner *sIL1RAP* was downregulated by insulin. However, normal human serum sIL1RAP concentration is approximately 300 ng/mL (25), suggesting a similarly high sIL1RAP level also in the placenta. Therefore, the effect of *sST2* upregulation is unlikely to be limited by the minimal decrease of *sIL1RAP* expression on insulin or the combined treatment.

Inflammation is tightly controlled during all stages of pregnancy; however, excessive, insufficient, delayed, or early-onset maternal inflammatory responses are linked to adverse pregnancy outcomes. Accordingly, low levels of IL-33 and/or high levels of sST2 were shown in the serum or placental tissue of preeclampsia patients (26‐32), while both IL-33 and sST2 concentrations have been increased in the serum of gestational diabetes patients (33). Furthermore, altered decidual expression of IL-33/sST2/ST2L was also suggested to play a key role in recurrent pregnancy loss and preterm labor (5, 6). These results are in line with the pathophysiological mechanisms observed during pregnancy in mice with maternal IL-33 deficiency (10).

Several lines of evidence suggest that insulin resistance and compensatory hyperinsulinemia, in addition to hyperandrogenism, play a key role in the pathophysiology of pregnancy complications, including gestational hypertension, preeclampsia, miscarriage, and recurrent pregnancy loss in women with obesity and PCOS (11‐16). We and others have previously reported that androgens and insulin alter the expression of several key endometrial receptivity genes/decidual markers in decidualized human ESCs *in vitro*, in addition to the modulation of the morphological characteristics and function of these cells (17, 34‐38). We hypothesize that the excessive upregulation of sST2 secretion from decidual stromal cells by high levels of androgens and insulin might pathologically downregulate uteroplacental IL-33 signaling, leading to defects of key processes before and during pregnancy, including decidualization, trophoblast invasion, and vascular remodeling. Therefore, the upregulation of sST2 secretion, in addition to the modulation of the expression of several key receptivity genes in the uteroplacental unit by androgens and insulin, might explain, at least partially, the association of hyperandrogenism and hyperinsulinemia and the aforementioned pregnancy complications. This hypothesis, in addition to the molecular mechanism of the combined action of DHT and insulin on the expression of sST2, needs to be further explored. Furthermore, our findings suggest that, similarly to other diseases (39‐43), targeting the IL33/ST2 signaling pathway could be a promising strategy to promote successful pregnancy outcomes.

The limitations of this study are the restricted number of endometrial samples and the interindividual variation of the results that might have led to type II error (false-negative results). Furthermore, the women had regular menstrual cycles but not all had been pregnant and therefore fertility was not proven. We consider the use of primary ESCs instead of a cell line a strength of the study.

Our observation that insulin and DHT upregulate sST2 secretion from primary human ESCs also leads to several new questions. For example, significant biological differences have been shown between ESCs obtained from PCOS patients vs control individuals, including an altered response to *in vitro* decidualization and DHT treatment (38, 44, 45). Furthermore, a selective metabolic signaling defect of the insulin and insulin-like growth factor-1 receptors was detected in skin fibroblasts (in addition to several other tissues) of PCOS patients (46, 47), suggesting that such a defect might also be present in the ESCs of women with PCOS. Similarly, insulin signaling defects can also be detected in tissues of patients with other types of insulin resistance (48). Therefore, ESCs obtained from these patient groups should also be studied. Furthermore, our findings need to be validated by analyzing the expression of the components of the IL-33 signaling pathway in endometrial and placental tissues of women with high insulin and/or androgen levels. Finally, to our best knowledge, this is the first report in any biological system that androgens and insulin can modulate IL-33 signaling, which raises the question whether the phenomenon is unique for ESCs, or it can also be detected in other cell and tissue types.

## Data Availability

A link to the supplementary data (Supplementary Table S1 and Supplementary Figs. S1-S3) is included in the references (20). Data sets generated during the present study are not publicly available but are available from the corresponding author on reasonable request.

## Abbreviations

cDNA: complementary DNA
db-cAMP: dibutyryl–cyclic adenosine monophosphate
DHT: dihydrotestosterone
ESCs: endometrial stromal cells
IL1RAP: IL-1 receptor accessory protein
IL1RL1: interleukin 1 receptor like 1
IL-33: interleukin 33
MPA: medroxyprogesterone-17-acetate
NK cells: natural killer cells
PCOS: polycystic ovary syndrome
sIL1RAP: soluble IL1RAP
sST2: soluble IL-33 receptor
ST2: IL-33 receptor
ST2L: long transmembrane form of IL-33 receptor

## References

[1] Kakkar R, Lee RT. The IL-33/ST2 pathway: therapeutic target and novel biomarker. Nat Rev Drug Discov. 2008;7(10):827‐840. 18827826 10.1038/nrd2660 PMC4277436

[2] Liew FY, Girard JP, Turnquist HR. Interleukin-33 in health and disease. Nat Rev Immunol. 2016;16(11):676‐689. 27640624 10.1038/nri.2016.95

[3] Palmer G, Lipsky BP, Smithgall MD, et al The IL-1 receptor accessory protein (AcP) is required for IL-33 signaling and soluble AcP enhances the ability of soluble ST2 to inhibit IL-33. Cytokine. 2008;42(3):358‐364. 18450470 10.1016/j.cyto.2008.03.008

[4] Vento-Tormo R, Efremova M, Botting RA, et al Single-cell reconstruction of the early maternal-fetal interface in humans. Nature. 2018;563(7731):347‐353. 30429548 10.1038/s41586-018-0698-6 PMC7612850

[5] Huang B, Faucette AN, Pawlitz MD, et al Interleukin-33-induced expression of PIBF1 by decidual B cells protects against preterm labor. Nat Med. 2017;23(1):128‐135. 27918564 10.1038/nm.4244 PMC5512431

[6] Salker MS, Nautiyal J, Steel JH, et al Disordered IL-33/ST2 activation in decidualizing stromal cells prolongs uterine receptivity in women with recurrent pregnancy loss. PLoS One. 2012;7(12):e52252. 23300625 10.1371/journal.pone.0052252 PMC3531406

[7] Fock V, Mairhofer M, Otti GR, et al Macrophage-derived IL-33 is a critical factor for placental growth. J Immunol. 2013;191(7):3734‐3743. 23997215 10.4049/jimmunol.1300490

[8] Hu WT, Huang LL, Li MQ, Jin LP, Li DJ, Zhu XY. Decidual stromal cell-derived IL-33 contributes to th2 bias and inhibits decidual NK cell cytotoxicity through NF-kappaB signaling in human early pregnancy. J Reprod Immunol. 2015;109:52‐65. 25712540 10.1016/j.jri.2015.01.004

[9] Hu WT, Li MQ, Liu W, Jin LP, Li DJ, Zhu XY. IL-33 enhances proliferation and invasiveness of decidual stromal cells by up-regulation of CCL2/CCR2 via NF-kappaB and ERK1/2 signaling. Mol Hum Reprod. 2014;20(4):358‐372. 24344240 10.1093/molehr/gat094

[10] Valero-Pacheco N, Tang EK, Massri N, et al Maternal IL-33 critically regulates tissue remodeling and type 2 immune responses in the uterus during early pregnancy in mice. Proc Natl Acad Sci U S A. 2022;119(35):e2123267119. 10.1073/pnas.2123267119 PMC9436313 35994660

[11] Acromite MT, Mantzoros CS, Leach RE, Hurwitz J, Dorey LG. Androgens in preeclampsia. Am J Obstet Gynecol. 1999;180(1 Pt 1):60‐63. 9914579 10.1016/s0002-9378(99)70150-x

[12] Hauth JC, Clifton RG, Roberts JM, et al Maternal insulin resistance and preeclampsia. Am J Obstet Gynecol. 2011;204(4):327 e1‐6. 10.1016/j.ajog.2011.02.024 PMC3127262 21458622

[13] Kumar S, Gordon GH, Abbott DH, Mishra JS. Androgens in maternal vascular and placental function: implications for preeclampsia pathogenesis. Reproduction. 2018;156(5):R155‐RR67. 30325182 10.1530/REP-18-0278 PMC6198264

[14] Parretti E, Lapolla A, Dalfra M, et al Preeclampsia in lean normotensive normotolerant pregnant women can be predicted by simple insulin sensitivity indexes. Hypertension. 2006;47(3):449‐453. 16446386 10.1161/01.HYP.0000205122.47333.7f

[15] Makieva S, Saunders PT, Norman JE. Androgens in pregnancy: roles in parturition. Hum Reprod Update. 2014;20(4):542‐559. 24643344 10.1093/humupd/dmu008 PMC4063701

[16] Okon MA, Laird SM, Tuckerman EM, Li TC. Serum androgen levels in women who have recurrent miscarriages and their correlation with markers of endometrial function. Fertil Steril. 1998;69(4):682‐690. 9548158 10.1016/s0015-0282(98)00007-7

[17] Ujvari D, Jakson I, Babayeva S, et al Dysregulation of In Vitro decidualization of human endometrial stromal cells by insulin via transcriptional inhibition of forkhead box protein O1. PLoS One. 2017;12(1):e0171004. 28135285 10.1371/journal.pone.0171004 PMC5279782

[18] Grino PB, Griffin JE, Wilson JD. Testosterone at high concentrations interacts with the human androgen receptor similarly to dihydrotestosterone. Endocrinology. 1990;126(2):1165‐1172. 2298157 10.1210/endo-126-2-1165

[19] Swerdloff RS, Wang C. Dihydrotestosterone: a rationale for its use as a non-aromatizable androgen replacement therapeutic agent. Baillieres Clin Endocrinol Metab. 1998;12(3):501‐506. 10332569 10.1016/s0950-351x(98)80267-x

[20] Salamon D, Ujvari D, Hellberg A, Hirschberg AL. Supplementary data for DHT and insulin up-regulate secretion of the soluble decoy receptor of IL-33 from decidualized endometrial stromal cells. Figshare. 2023. 10.6084/m9.figshare.24291391 PMC10681354 37972259

[21] ggplot2: Elegant Graphics for Data Analysis [Program]. 2nd. version. Springer International Publishing: Imprint: Springer, 2016.

[22] Mor G, Aldo P, Alvero AB. The unique immunological and microbial aspects of pregnancy. Nat Rev Immunol. 2017;17(8):469‐482. 28627518 10.1038/nri.2017.64

[23] Gellersen B, Brosens JJ. Cyclic decidualization of the human endometrium in reproductive health and failure. Endocr Rev. 2014;35(6):851‐905. 25141152 10.1210/er.2014-1045

[24] Wilczynski JR . Th1/Th2 cytokines balance–yin and yang of reproductive immunology. Eur J Obstet Gynecol Reprod Biol. 2005;122(2):136‐143. 15893871 10.1016/j.ejogrb.2005.03.008

[25] Smith DE, Hanna R, Della F, et al The soluble form of IL-1 receptor accessory protein enhances the ability of soluble type II IL-1 receptor to inhibit IL-1 action. Immunity. 2003;18(1):87‐96. 12530978 10.1016/s1074-7613(02)00514-9

[26] Chen H, Zhou X, Han TL, Baker PN, Qi H, Zhang H. Decreased IL-33 production contributes to trophoblast cell dysfunction in pregnancies with preeclampsia. Mediators Inflamm. 2018;2018:9787239. 29736154 10.1155/2018/9787239 PMC5875049

[27] Granne I, Southcombe JH, Snider JV, et al ST2 And IL-33 in pregnancy and pre-eclampsia. PLoS One. 2011;6(9):e24463. 21949719 10.1371/journal.pone.0024463 PMC3174956

[28] Romero R, Chaemsaithong P, Tarca AL, et al Maternal plasma-soluble ST2 concentrations are elevated prior to the development of early and late onset preeclampsia—a longitudinal study. J Matern Fetal Neonatal Med. 2018;31(4):418‐432. 28114842 10.1080/14767058.2017.1286319 PMC5581264

[29] Sasmaya PH, Khalid AF, Anggraeni D, Irianti S, Akbar MR. Differences in maternal soluble ST2 levels in the third trimester of normal pregnancy versus preeclampsia. Eur J Obstet Gynecol Reprod Biol X. 2022;13:100140. 34917932 10.1016/j.eurox.2021.100140 PMC8669363

[30] Stampalija T, Chaiworapongsa T, Romero R, et al Maternal plasma concentrations of sST2 and angiogenic/anti-angiogenic factors in preeclampsia. J Matern Fetal Neonatal Med. 2013;26(14):1359‐1370. 23488689 10.3109/14767058.2013.784256 PMC6333087

[31] Xiang Q, Chen Y, Gu X, Yang Y, Wang Y, Zhao Y. The correlation between maternal serum sST2, IL-33 and NT-proBNP concentrations and occurrence of pre-eclampsia in twin pregnancies: A longitudinal study. J Clin Hypertens (Greenwich). 2022;24(11):1516‐1523. 36149818 10.1111/jch.14579 PMC9659875

[32] Kanninen T, Jung E, Gallo DM, et al Soluble suppression of tumorigenicity-2 in pregnancy with a small-for-gestational-age fetus and with preeclampsia. J Matern Fetal Neonatal Med. 2023;36(1):2153034. 36521862 10.1080/14767058.2022.2153034 PMC10291739

[33] Fan W, Kang W, Li T, et al Interleukin-33 and its receptor soluble suppression of tumorigenicity 2 in the diagnosis of gestational diabetes mellitus. Int J Clin Pract. 2021;75(12):e14944. 34605145 10.1111/ijcp.14944

[34] Hirschberg AL, Jakson I, Graells Brugalla C, Salamon D, Ujvari D. Interaction between insulin and androgen signalling in decidualization, cell migration and trophoblast invasion in vitro. J Cell Mol Med. 2021;25(20):9523‐9532. 34463022 10.1111/jcmm.16892 PMC8505820

[35] Ujvari D, Graells Brugalla C, Hirschberg AL. Dihydrotestosterone potentiates insulin to up-regulate prokineticin-1 in decidualizing human endometrial stromal cells. J Cell Mol Med. 2020;24(5):3242‐3245. 31991505 10.1111/jcmm.14923 PMC7077604

[36] Ujvari D, Jakson I, Oldmark C, et al Prokineticin 1 is up-regulated by insulin in decidualizing human endometrial stromal cells. J Cell Mol Med. 2018;22(1):163‐172. 28782224 10.1111/jcmm.13305 PMC5742737

[37] Kajihara T, Tanaka K, Oguro T, et al Androgens modulate the morphological characteristics of human endometrial stromal cells decidualized in vitro. Reprod Sci. 2014;21(3):372‐380. 23885104 10.1177/1933719113497280

[38] Khatun M, Meltsov A, Lavogina D, et al Decidualized endometrial stromal cells present with altered androgen response in PCOS. Sci Rep. 2021;11(1):16287. 34381107 10.1038/s41598-021-95705-0 PMC8357821

[39] Altara R, Ghali R, Mallat Z, Cataliotti A, Booz GW, Zouein FA. Conflicting vascular and metabolic impact of the IL-33/sST2 axis. Cardiovasc Res. 2018;114(12):1578‐1594. 29982301 10.1093/cvr/cvy166

[40] Braun H, Afonina IS, Mueller C, Beyaert R. Dichotomous function of IL-33 in health and disease: from biology to clinical implications. Biochem Pharmacol. 2018;148:238‐252. 29309756 10.1016/j.bcp.2018.01.010

[41] Chan BCL, Lam CWK, Tam LS, Wong CK. IL33: Roles in allergic inflammation and therapeutic perspectives. Front Immunol. 2019;10:364. 30886621 10.3389/fimmu.2019.00364 PMC6409346

[42] Shakerian L, Kolahdooz H, Garousi M, et al IL-33/ST2 axis in autoimmune disease. Cytokine. 2022;158:156015. 36041312 10.1016/j.cyto.2022.156015

[43] Sun Y, Wen Y, Wang L, et al Therapeutic opportunities of interleukin-33 in the central nervous system. Front Immunol. 2021;12:654626. 34079543 10.3389/fimmu.2021.654626 PMC8165230

[44] Piltonen TT, Chen JC, Khatun M, et al Endometrial stromal fibroblasts from women with polycystic ovary syndrome have impaired progesterone-mediated decidualization, aberrant cytokine profiles and promote enhanced immune cell migration in vitro. Hum Reprod. 2015;30(5):1203‐1215. 25750105 10.1093/humrep/dev055 PMC4400200

[45] Younas K, Quintela M, Thomas S, et al Delayed endometrial decidualisation in polycystic ovary syndrome; the role of AR-MAGEA11. J Mol Med (Berl). 2019;97(9):1315‐1327. 31256208 10.1007/s00109-019-01809-6 PMC6713698

[46] Book CB, Dunaif A. Selective insulin resistance in the polycystic ovary syndrome. J Clin Endocrinol Metab. 1999;84(9):3110‐3116. 10487672 10.1210/jcem.84.9.6010

[47] Dunaif A, Xia J, Book CB, Schenker E, Tang Z. Excessive insulin receptor serine phosphorylation in cultured fibroblasts and in skeletal muscle. A potential mechanism for insulin resistance in the polycystic ovary syndrome. J Clin Invest. 1995;96(2):801‐810. 7635975 10.1172/JCI118126 PMC185266

[48] Petersen MC, Shulman GI. Mechanisms of insulin action and insulin resistance. Physiol Rev. 2018;98(4):2133‐2223. 30067154 10.1152/physrev.00063.2017 PMC6170977

